# A nationwide survey of healthcare personnel’s attitude, knowledge, and interest toward renal supportive care in Taiwan

**DOI:** 10.7717/peerj.3540

**Published:** 2017-07-07

**Authors:** Hung-Bin Tsai, Chia-Ter Chao, Jenq-Wen Huang, Ray-E Chang, Kuan-Yu Hung, COhort of GEriatric Nephrology in NTUH (COGENT) study group

**Affiliations:** 1 Division of Hospital Medicine, Department of Internal Medicine, National Taiwan University Hospital, Taipei, Taiwan; 2 Institute of Health Policy and Management, College of Public Health, National Taiwan University, Taipei, Taiwan; 3 Department of Internal Medicine, College of Medicine, National Taiwan University, Taipei, Taiwan; 4 Department of Medicine, National Taiwan University Hospital Jinshan Branch, New Taipei City, Taiwan; 5 Division of Nephrology, Department of Internal Medicine, National Taiwan University Hospital, Taipei, Taiwan; 6 Department of Internal Medicine, National Taiwan University Hospital Hsin-Chu Branch, Hsin-Chu County, Taiwan

**Keywords:** End-stage renal disease, Dialysis, Medical center, Renal supportive care, Taiwan, Cross-sectional studies, Palliative care, Allied health occupations, Medical education

## Abstract

**Background:**

Renal supportive care (RSC) is an important option for elderly individuals reaching end-stage renal disease; however, the frequency of RSC practice is very low among Asian countries. We evaluated the attitude, the knowledge, and the preference for specific topics concerning RSC among participants who worked in different medical professions in Taiwan.

**Methods:**

A cross-sectional questionnaire-based survey was employed. Healthcare personnel (*N* = 598) who were involved in caring for end-stage renal disease patients at more than 40 facilities in Taiwan participated in this study. Participants were asked about their motivation for learning about RSC, the topics of RSC they were most and least interested in, their willingness to provide RSC, and to rate their knowledge and perceived importance of different topics.

**Results:**

The vast majority of respondents (81.9%) were self-motivated about RSC, among whom nephrologists (96.8%) and care facilitators (administrators/volunteers) (45%) exhibited the highest and the least motivation, respectively (*p* < 0.01). Overall, respondents indicated that they had adequate knowledge about the five pre-specified RSC topics between medical professions (*p* = 0.04). Medical professions and institutional size exerted significant influence on the willingness to provide RSC.

**Conclusions:**

Our results facilitate the understanding of the knowledge and attitude toward different RSC topics among varied medical professions, and can guide the design of RSC education content for healthcare personnel.

## Introduction

The accelerated pace of population aging constitutes an important public health concern, as the care for elderly with multi-morbidities becomes more complicated with aging-associated organ degeneration (Bähler et al., 2015; Chao et al., 2015a). Among the comorbidities accompanying advanced age, chronic kidney disease (CKD) is particularly important; the prevalence of CKD among elderly can be three to six fold higher than it is in younger individuals (Zhang & Rothenbacher, 2008). The incidence of end-stage renal disease (ESRD) among elderly also rises succinctly over time, and ESRD elderly are increasingly started on renal replacement therapy (RRT) (United States Renal Data System, 2016). However, the increasing functional deficits, aggravating symptomatology from multimorbidity, and the emergence of degenerative syndromes including frailty, contributes to the uncertainty about the benefits and harms of RRT in this population (Chao et al., 2015b; Chao & Chiang, 2016; Chao, Huang & Chiang, 2016a; Kallenberg et al., 2016). A considerable degree of frail phenotypic heterogeneity exists for elderly with ESRD and often has poor clinical outcomes (Chao & Huang, 2015; Chao, Chan & Huang, 2017); although the prediction of short-term mortality among elderly with ESRD is feasible, accurate estimates of individual survival are nonetheless difficult (Moss et al., 2008; Couchoud et al., 2009). Furthermore, a large proportion of elderly with ESRD have a heavy symptom burden, which is not necessarily ameliorable by dialysis (Rivara et al., 2015). Besides, elderly patients tend to have more non-medical barriers than younger patients do, such as variable family support, economic limitations, transportation difficulties, and life-goal differences (Kurella Tamura, 2009). The ambiguity in projecting survival, the possibility of prolonged suffering, and poorer quality of life necessitates a reconciling between whether to initiate or continue RRT among elderly with ESRD or not.

After weighing the RRT risks against the benefits, elderly with ESRD may choose to receive renal supportive care (RSC), or maximal conservative management without dialysis. Those who agree to RSC receive the same care as they do in earlier stages of CKD, with particular emphasis on symptomatic and holistic care (Chao et al., 2016b). There are significant geographical variations in the prevalence of RSC practice. The practice of RSC in East Asia is considerably uncommon compared to that in Western countries. The prevalence of dialysis withdrawal was significantly lower in East Asia than it was in the US and European countries (Fissell et al., 2005). In Taiwan, nephrologists and other affiliated staff infrequently promote RSC, and dialysis withdrawal is only offered during exceptional circumstances (Chao et al., 2016b). The reason for this lower RSC adoption rate is currently unclear, and none of the existing studies evaluated the perception of RSC among healthcare personnel in Taiwan, despite its extremely high incidence and prevalence of ESRD. In this study, we conducted a nationwide survey to evaluate the attitude and the knowledge of RSC among a large group of healthcare personnel involved in ESRD patient care.

**Figure 1:**
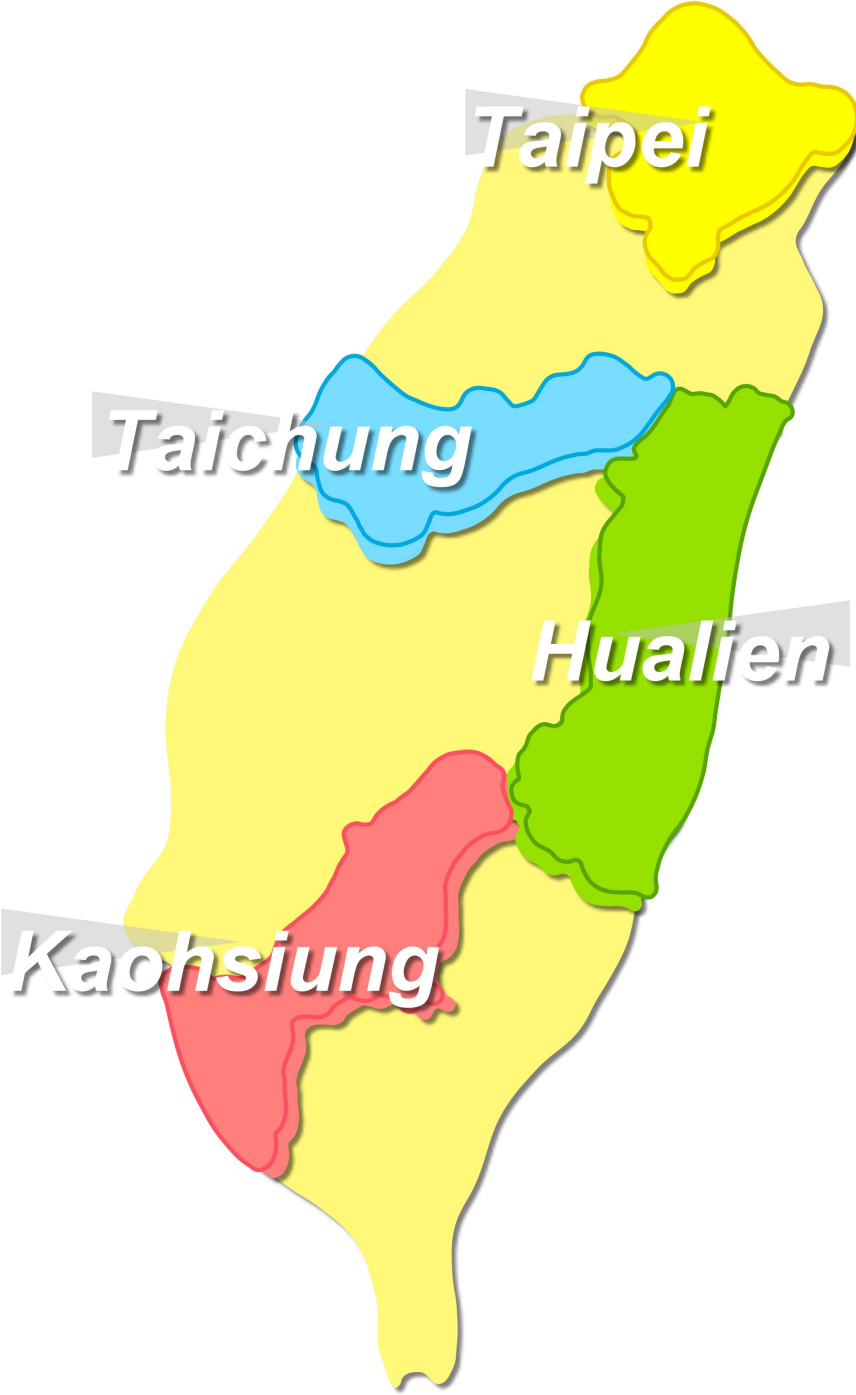
Geographical sites with continuous medical education courses being held in Taiwan.

## Methods

A questionnaire-based survey was conducted among all healthcare personnel attending continued medical education (CME) courses focusing on RSC between January–April 2016 in four major Taiwan cities (north, Taipei; central, Taichung; south, Kaohsiung; and east, Hualien) (Fig. 1). The CME courses were jointly provided by the Taiwan Society of Nephrology and the Taiwan Society of Hospice and Palliative care, aiming to enhance the awareness and the understanding of RSC among practitioners. The courses addressed topics including but not limited to the inception, the evolution, and the changing pattern of RSC in global and domestic arenas, the laws that bind the RSC concept and practice to healthcare personnel in Taiwan, the terminology and clinical implications of RSC, inter-disciplinary actions and collaborations for RSC, and experiences sharing between institutions.

Participants came from more than 40 hospitals and stand-alone dialysis clinics in Taiwan, and those who successfully completed all the courses would be awarded points required for their certificate renewal. The questionnaire was distributed before the curriculum and the results served as the basis of this report. Participants were classified according to their medical professions (physicians, dialysis nurses, case managers, or care facilitators (administrators or volunteers)), residential areas, size of their parent institution, and the duration of experiences involved in ESRD patient care. The volunteers we described in this study denote “hospital volunteers” or “medical volunteers”, who received training by hospitals or non-for-profit organizations before service, worked without payment in the healthcare settings.

The questionnaire consisted of 20 items divided into two main categories; the first category addressed the background information of participants (including medical professions, the size of the affiliated institution, and the ESRD patient-care practice duration); motivation for learning about RSC; the topics that they were most or least interested and whether they would provide RSC for appropriate candidates during their practice (in a multiple choice format). The other category evaluated one’s knowledge basis perceived topic importance using ratings on a 5-point Likert scale (5 = *most important* and 1 = *least important*). These scales were applied to 5 pre-specified core topics after literature review: RSC history introduction, local regulations and laws for RSC, indications of RSC and advance care planning (ACP), symptom control and palliative dialysis, and RSC practice in clinical scenarios using case vignettes. These topics were chosen based on the consensus recommended by a panel of experts from the Taiwan Society of Nephrology before the study commencement. Each item was further modified and rephrased after review by external experts with expertise in subject content. All identifying information of participants was anonymized. This study was approved by the Institutional Review Board of the National Taiwan University Hospital (NO. 201512157RINC) and was conducted in accordance with the declaration of Helsinki. Verbal informed consent was obtained from all participants during the distribution of study questionnaires, and was facilitated by the same group of research assistants throughout the study period.

All statistical comparisons were made using SPSS 18.0 (Chicago, IL, USA). Continuous variables were compared using independent *t*-test, while categorical variables were compared using Chi-square tests. Comparisons between three or more groups were conducted by an analysis of variance (ANOVA). A two-sided *p*-value of less than 0.05 was considered statistically significant.

## Results

Eight-hundred questionnaires were distributed and 598 (response rate: 74.8%) were returned. The respondents’ clinical features are listed in Table 1. Among the physician participants, one-third were nephrology or palliative care training fellows, while others were nephrologists or palliative care specialists.

**Table 1:** Clinical features of the respondents.

Features	Descriptions
*Medical professions*	
Nephrologists (and trainees)	31 (5.2%)
Palliative care specialists (and trainees)	10 (1.7%)
Dialysis nurses orcase managers	497 (83.1%)
Care facilitators (administrators or volunteers)	60 (10%)
*Geographic residence*	
Northern part	109 (18.2%)
Central part	225 (37.6%)
Southern part	164 (27.4%)
Eastern part	100 (16.7%)
*Institutional size*	
Academic medical centers	96 (16.1%)
Metropolitan hospitals	153 (25.6%)
Local community hospitals	109 (18.2%)
Dialysis clinics	174 (29.1%)
Primart care clinics	66 (11%)
ESRD patient care practice duration (years)	16.1 ± 8.3

**Notes.**

ESRD end-stage renal disease

### Motivations about learning RSC among healthcare personnel and the influencing factors

Many respondents (81.9%) were self-motivated about RSC, while 18.1% attended the RSC courses due to the requirement of their institutes. Respondents from southern Taiwan were significantly more likely to be self-motivated about RSC (86%) compared to the others, while those from east Taiwan were the least likely (76%) (*p* < 0.01). Nephrologists exhibited the highest motivation for learning about RSC (96.8%), followed by dialysis nurses or case managers (85.5%), and palliative care specialists (80%), while care facilitators had significantly lower motivation for learning RSC (45%) (*p* < 0.01). In addition, institutional size exerted significant influence on the respondent’s motivation to learn about RSC; those from academic medical centers exhibited the highest motivation (90.6%), followed by metropolitan hospitals (86.9%), local community hospitals (87.2%), dialysis clinics (84.5%), and primary care clinics (42.4%) (*p* < 0.01).

### RSC topics of interest to healthcare personnel

Among the five pre-specified topics, 26.5% respondents were most interested in the topic “indications of RSC, how to select the appropriate candidates for RSC”, and followed by the topic “symptom control and palliative dialysis” (21.7%), and “the introduction to RSC history” (18.9%). Only 15.4% respondents chose “RSC practice case vignettes” as the most interesting topic. On the other hand, the least interesting topic was “local regulations and laws for RSC” (35.6%). Duration of practice of caring for dialysis patients (*p* =0.79) or institutional size (*p* =0.43) did not significantly influence the topic gaining the most interest among respondents; however, the choice differed significantly between different medical professions. Nephrologists (30.8%), palliative care specialists (66.7%), and dialysis nurses/case managers (25.7%) were most interested in “indications of RSC and ACP”, while care facilitators (40.9%) were most interested in “introduction to RSC history” (*p* =0.04). The second most topics for nephrologists, palliative care specialists, and dialysis nurses/case managers were “introduction to RSC history” (26.9%), “local regulations and laws for RSC” (22.2%), and “symptom control and palliative dialysis” (22.3%), respectively (*p* =0.04).

Overall, respondents indicated that they had adequate knowledge about the five topics (Table 2). Similarly, respondents felt that the five RSC topics were of near equal importance (mean ratings for RSC history introduction: 4.13 ± 1.36, local regulations/laws: 4.22 ± 1.12, RSC indications/ACP: 4.23 ± 1.14, symptom control/palliative dialysis: 4.23 ± 1.2, and RSC practice case vignettes: 4.02 ± 1.5). Significant difference was found between respondents of different medical professions regarding knowledge or perceived importance of each topic (Table 2). However, those with practice for more than 10 years reported significantly less adequate knowledge about RSC history introduction (*p* = 0.02) and RSC practice case vignettes (*p* < 0.01), and placed less importance on RSC case vignettes (*p* < 0.01). Furthermore, respondents from local community hospitals reported having the least knowledge in RSC history introduction (3.7 ± 1.64), followed by those from dialysis clinics (3.95 ± 1.29), and metropolitan hospitals (3.99 ± 1.29), compared to those from medical centers (4.3 ± 1.01) (*p* = 0.03) (Table 3). Respondents from local community hospitals also thought that the introduction to RSC history was least important compared to those from other institutes (*p* = 0.03). On the other hand, respondents from medical centers placed the most emphasis on the introduction of RSC history (*p* = 0.03), the indications of RSC/ACP (*p* = 0.047), symptom control and palliative dialysis (*p* = 0.04), and RSC practice case vignettes (*p* = 0.02) than the other respondents did (Table 3).

**Table 2:** Ratings of respondents with different medical professions about their attitude and knowledge regarding renal supportive care.

Topics	All (*n* = 598)	Nephrologists (*n* = 31)	Palliative care specialists (*n* = 10)	RN and case managers (*n* = 497)	^a^Others (*n* = 60)	*P* value
*Knowledge adequacy*						
Introduction about RSC history	3.98 ± 1.35	3.84 ± 1.64	4.2 ± 1.55	3.98 ± 1.31	4.1 ± 1.49	0.78
Local regulation and laws for RSC	4.1 ± 1.14	3.84 ± 1.61	4.3 ± 0.68	4.1 ± 1.08	4.25 ± 1.4	0.4
Indications of RSC and ACP	4.07 ± 1.18	3.9 ± 1.51	4.2 ± 0.79	4.08 ± 1.12	4.08 ± 1.5	0.85
Symptom contol and palliative dialysis	4.08 ± 1.24	3.97 ± 1.45	4.1 ± 0.74	4.09 ± 1.19	4.07 ± 1.58	0.96
RSC practice in clinical scenarios using case vignettes	3.9 ± 1.52	3.68 ± 1.72	4.4 ± 0.84	3.9 ± 1.48	3.92 ± 1.74	0.62
*Perceived importance*						
Introduction about RSC history	4.13 ± 1.36	4.1 ± 1.68	4.2 ± 1.62	4.12 ± 1.32	4.15 ± 1.49	0.99
Local regulation and laws for RSC	4.22 ± 1.12	4 ± 1.65	4.7 ± 0.48	4.22 ± 1.06	4.25 ± 1.39	0.38
Indications of RSC and ACP	4.23 ± 1.14	4 ± 1.55	4.6 ± 0.52	4.26 ± 1.07	4.1 ± 1.48	0.35
Symptom contol and palliative dialysis	4.23 ± 1.2	4.19 ± 1.49	4.4 ± 0.7	4.25 ± 1.14	4.1 ± 1.58	0.79
RSC practice in clinical scenarios using case vignettes	4.02 ± 1.5	3.81 ± 1.78	4.5 ± 0.71	4.04 ± 1.46	3.93 ± 1.74	0.59

**Notes.**

Data are expressed as mean ± standard deviation for continuous variables.

a Others included administrators and volunteers.

ACP advanced care planning RN registered nurse RSC renal supportive care

**Table 3:** Ratings of respondents from different institutional levels about their attitude and knowledge regarding renal supportive care.

Topics	Medical Centers (*n* = 96)	Metropolitan Hospitals (*n* = 153)	Community Hospitals (*n* = 109)	Dialysis Clinics (*n* = 174)	Primary care clinics (*n* = 66)	*P* value
*Knowledge adequacy*						
Introduction about RSC history	4.3 ± 1.01	3.99 ± 1.29	3.7 ± 1.64	3.95 ± 1.29	4.08 ± 1.44	0.03
Local regulation and laws for RSC	4.35 ± 0.92	4.02 ± 1.17	4.01 ± 1.23	4.07 ± 1.09	4.18 ± 1.34	0.16
Indications of RSC and ACP	4.36 ± 0.82	4.03 ± 1.2	3.95 ± 1.26	4.03 ± 1.15	4.06 ± 1.44	0.12
Symptom contol and palliative dialysis	4.29 ± 1.03	4.01 ± 1.27	3.99 ± 1.32	4.05 ± 1.19	4.14 ± 1.42	0.4
RSC practice in clinical scenarios using case vignettes	4.23 ± 1.25	3.78 ± 1.5	3.68 ± 1.72	3.9 ± 1.51	4.05 ± 1.51	0.08
*Perceived importance*						
Introduction about RSC history	4.46 ± 0.98	4.12 ± 1.27	3.83 ± 1.7	4.13 ± 1.33	4.11 ± 1.44	0.03
Local regulation and laws for RSC	4.48 ± 0.91	4.18 ± 1.1	4.08 ± 1.24	4.22 ± 1.08	4.2 ± 1.33	0.14
Indications of RSC and ACP	4.54 ± 0.75	4.21 ± 1.15	4.11 ± 1.25	4.22 ± 1.11	4.08 ± 1.43	0.047
Symptom contol and palliative dialysis	4.54 ± 0.86	4.22 ± 1.22	4.05 ± 1.32	4.21 ± 1.17	4.18 ± 1.42	0.04
RSC practice in clinical scenarios using case vignettes	4.4 ± 1.17	3.99 ± 1.46	3.7 ± 1.73	4.03 ± 1.51	4.08 ± 1.51	0.02

**Notes.**

Data are expressed as mean ± standard deviation for continuous variables.

ACP advanced care planning RSC renal supportive care

### Willingness to provide RSC to patients in the future

Overall, 87.1% respondents expressed their willingness to provide RSC during practice. Duration of ESRD patient care or geographic residence did not influence the preference in providing RSC (*p* = 0.09 and 0.06, respectively). On the contrary, professions and institutional size significantly affected the preference in providing RSC. Palliative care specialists had the highest willingness to provide RSC in the future (100%), followed by nephrologists (96.8%), and dialysis nurses/case managers (91.8%), while administrators/volunteers had the lowest willingness (41.7%) (*p* < 0.05). Those who worked in dialysis clinics exhibited the highest willingness to provide RSC (93.1%), followed by metropolitan hospitals (92.85), medical centers (91.7%), and local community hospitals (91.7%), while those from primary care clinics exhibited the lowest willingness (43.9%) (*p* < 0.01).

## Discussion

Findings from this study disclose that among a large group of experienced healthcare personnel in Taiwan, more than 80% were self-motivated about learning RSC, and they were most interested in topics including the indications of RSC/ACP and symptom control/palliative dialysis. On average, respondents felt that their knowledge about the five RSC topics was adequate, and these topics were found to be of near equal importance. Nearly 90% were willing to provide RSC during their practice. Geographic residences, medical professions, and institutional sizes significantly influenced attitude toward learning RSC and the relevant knowledge, the topic of interest, and the willingness to provide RSC. These findings might serve as important bases for designing appropriate RSC course content and for effectively directing educational resources toward suitable candidates.

Literature suggests that patients with ESRD have little access to hospice and palliative care service compared to those with cancer or chronic life-threatening illnesses, and healthcare staffs are also ill prepared for providing RSC (Berzoff, Swantkowski & Cohen, 2008; Culp et al., 2016). In a cross-sectional survey, Combs and colleagues reported that 95% of US nephrology trainees felt that it was moderately to very important to learn how to provide RSC; however, their knowledge in this field was insufficient to meet the requirements (Combs et al., 2015). Another European survey similarly disclosed that 70–74% of nephrologists did not acquaint themselves with palliative care (Van Biesen et al., 2015). These findings suggest that despite the awareness of its importance, nephrologists and their affiliated staffs are sub-optimally educated for incorporating RSC into their practice. This lack of proper RSC education might be related to the failure of identifying potential candidates, a low interest for specific topics within RSC, or the poor efficacy of existing educational techniques (Chung et al., 2016). Socio-cultural and geographic disparities also play a role in influencing the outcomes of RSC education (Chao et al., 2016b). Consequently, a comprehensive survey of the attitude, the knowledge, and the preferential issues for RSC among practitioners and their affiliated staffs is needed to optimize the educational outcomes. Results of this study provide important insights into factors that affect the staffs’ attitude, preferences, and willingness to provide RSC.

We observed a major discrepancy between the high willingness to provide RSC shown by this survey and the low prevalence of RSC practice in Taiwan. Despite our finding that 81.9% and 87.1% respondents reported high motivation to learn RSC and were willing to provide RSC in the future, respectively, a claim database registry identified that CKD patients were responsible for only 1% to 2% hospice/palliative reimbursement (Lai, Hsu & Huang, 2015). Several plausible reasons might be contributory. First, the decisions to provide RSC can be partially influenced by the healthcare reimbursement infrastructure (Schreibeis-Baum et al., 2016). If the compensation for continuing dialysis treatments potentially outnumbers that for providing RSC, the financial balance would dip toward the former (Van Biesen et al., 2015; Aldridge et al., 2016). This was supported by our finding that facility administrators, who were responsible for facility operation and financial stability, were significantly less motivated for learning RSC (45%) (*p* < 0.01) and less willing to provide RSC (43.9%) (*p* < 0.01) compared to the others (all ≧ 80%). Although nephrologists and dialysis nurses had high motivation and willingness, they could be constrained by the reimbursement limitations and act differently from their preferences. Second, as many as 40% of patients with advanced CKD and ESRD received medical care from care clinics instead of nephrologists, and the attitude toward RSC from staffs of these clinics might directly affect the patients’ decision-making processes (Tam-Tham et al., 2016). It has been found that community clinicians tend to attribute the responsibility of end-of-life discussions to palliative care teams or illness-specific specialists (Dunlay et al., 2015). We also found that respondents from primary clinics exhibited significantly lower motivation (42.4%; *p* < 0.01) and lower willingness (43.9%; *p* < 0.01) to provide RSC compared to those from other facilities. Finally, the respondents might be unfamiliar with the practical experience of RSC despite their positive attitude and aspiration for RSC. Judging our findings, care facilitators including facility administrators and staffs from primary care clinic might be important candidates for RSC education in Taiwan.

Studies addressing which parts of RSC are most interesting for nephrologists and primary care providers are not uncommon; however, reports focusing on other medical professions are rare. A recent survey of attitude for RSC showed that nephrologists were most likely to encounter difficulty in recognizing potential candidates and were more interested in this issue compared to other clinicians (Parvez et al., 2016). In this study, we similarly found that nephrologists were most interested in the indications of RSC; furthermore, we discovered that significant heterogeneity existed in the preference of RSC topics among different medical professions. Care facilitators were most interested in RSC history introduction instead of indications of RSC. It is likely that these personnel are not involved in patients’ medical care directly, and thus are more interested in the concept and outlining of RSC construct that in the practical aspects. This was also supported by our findings that staffs working in academic medical centers were more familiar with introduction to RSC history and more likely to feel that the practical aspects of RSC were important (Table 3), since medical centers in Taiwan are often pilots in introducing new therapeutic modalities and care pathways.

We demonstrated that institutional size and medical professions modified the attitude and preference in the provision of RSC; these findings have been reported before mostly in studies focusing on hospice care for non-ESRD patients. Lindley and colleagues identified that smaller- and medium-sized facilities were less likely to provide hospice care for at needed candidates, probably due to a lack of internal resources and the capabilities required (Lindley et al., 2013). Another large, nationally representative survey also showed that larger hospitals and teaching status are important organizational determinants of hospice service provision (White, Cochran & Patel, 2002). Our findings discovered that respondents exhibited progressively lower motivation to learn RSC from academic medical centers (highest) to dialysis clinics/primary care clinics (lowest) supports this institutional size-hospice provision relationship. In addition, the attitude toward the components of hospice/palliative care, such as candidate selection and practical (decision-making), often differ between patients, family members, and healthcare personnel, and diversity between medical professions also exists (Sprung et al., 2007). For example, studies have found that team-based decision making was considered more important among nurses than physicians, while physicians valued treatment-related quality of life changes stronger than other caregivers (Raijmakers et al., 2012). Additionally, nephrologists exhibited higher motivation to learn about RSC and higher willingness to provide RSC than dialysis nurses did (*p* < 0.01 for both comparisons), despite that both professions achieved >80% agreement in these questions. This might result from the fact that each medical profession holds its own responsibilities, is involved in patient care to different and plays a different role in care coordination (Raijmakers et al., 2012). In Taiwan, physicians tend to assume more responsibility for patient care than other professions, potentially leading to the finding that nephrologists exhibit higher willingness and motivation to provide such care.

We noted that the duration of care for ESRD patients also influenced the knowledge levels and the perceived importance about specific topics of RSC, including RSC history introduction and case vignettes. We propose that healthcare staffs with longer experience of ESRD care might be more familiar with the untold sufferings and the life stories of these patients, and have more time to ponder on the pros and cons of initiating/continuing dialysis, than the newcomers do. It is then reasonable that they place more emphasis on other issues of RSC rather than understanding the history of RSC and the real-world experiences of RSC.

It has been reported that patients with advanced CKD or ESRD have little access to RSC, and possess poor knowledge about their disease trajectory and the option of conservative care (Murray et al., 2006). Compared to those with metastatic malignancy, patients with advanced CKD or ESRD have comparable prevalence of symptom burden and severity (Saini et al., 2006), but they often rely upon nephrologists or other dialysis staff for symptom survey and for obtaining necessary information regarding RSC, such as advanced care planning and end-of-life discussions (Davison, 2010). More importantly, studies showed that more than 60% of ESRD patients regretted their decision to start dialysis (Davison, 2010), and CKD patients who received RSC might have better satisfaction with life compared to their companions who initiate dialysis (Da Silva-Gane et al., 2012). These issues exemplify the importance of understanding the need and wish of renal patients’ choices regarding their treatment plans, but from the perspectives of healthcare providers, substantial barriers, including system-level and individual-level ones exist, which preclude optimal interdisciplinary collaboration and RSC delivery (O’Hare et al., 2016). The situation is particularly serious in Taiwan, largely due to the lack of investigations into relevant obstacles. We previously established a local RSC program within a rural community in Taiwan (Chao et al., 2016b). In such program, we select suitable candidates based on a validated rating scale, and harness a 3-step and 4-phase protocol to provide RSC to these patients. Although this experience is not readily extrapolated to other parts of the country, physicians, dialysis nurses, and palliative care specialists involved in our established program all showed high enthusiasm and willingness to implement RSC, lending support to the findings of this survey. In this sense, our findings may serve to pinpoint some educational opportunities to address the promotion of RSC in this country.

## Limitation

The main limitations of this study include that this survey was conducted in only Taiwan, thereby limiting the generalizability of our findings, and the pre-specified five core topics of RSC, which could not cover all the important issues in this field. Since personnel who were interested in RSC tended to attend the CME course, it is possible that they would choose to answer the survey when they were asked to, reflecting the preferential selection of those with higher enthusiasm for RSC. In addition, the activity during which we distributed the questionnaires was held jointly by two professional societies in Taiwan, and CME points were offered exclusively to their members. It is expectable that physician participants of this survey were nephrologists or palliative care specialists, members of these societies, and extrapolation of our findings to other medical specialists might not be appropriate. The proportion of nephrologists participating in this survey was about 3%, also limiting the generalizability of these results. Reasons for the associations between medical professions, institution size, and knowledge of, attitude toward, and willingness of RSC cannot be known for sure in a cross-sectional questionnaire-based survey like this one. Furthermore, the age of respondents was not recorded at the time of study, rendering subgroup analyses based on different age groups impossible. Nonetheless, our study serves as an important attempt to understand the knowledge and attitude toward different components of RSC among different medical professions. For the first time, institutional size and duration of care experience were found to have important influences over the motivation, willingness, and RSC topic of interest among relevant staffs. These results can guide the subsequent design of education content for healthcare personnel involved in RSC.

##  Supplemental Information

10.7717/peerj.3540/supp-1 Supplemental Information 1 Supplementary file

10.7717/peerj.3540/supp-2 Supplemental Information 2 Questionnaire form

## Additional Information and Declarations

### Data Availability

The following information was supplied regarding data availability: The raw data has been supplied as a Supplemental File.
