# Supplementary material for: A nationwide survey of healthcare personnel’s attitude, knowledge, and interest toward renal supportive care in Taiwan
Source: PeerJ. 2017 Jul 7;5:e3540. doi: 10.7717/peerj.3540 (PMC5502085; doi:10.7717/peerj.3540)
Supplement: Supplemental Information 2 [file peerj-05-3540-s002.pdf]

一、教學意見: (請於個欄填寫您認為合適的數字)

說明:每欄依上述項目分別給分; 給分標準皆為 1 分至 5 分, **5 分為最高分**, 以整數遞減為 4 分、3 分、2 分至 1 分, **1 分最低**。

編號	課程名稱	講師	對課程的評值(on the seminar)			對講師的評值 (on the lecturer)			對自我的評值 (on the learner)				
			態度上<br>對安寧療護的體認	知識上<br>理念與思考的啟發	技術上<br>臨床與實務的運用	態度上<br>精神感召	知識上<br>專業知識	技術上<br>講解能力	態度上<br>投入意願程度	知識上<br>課程已知程度	知識上<br>課後增加程度	技術上<br>課程已知程度	技術上<br>課後增加程度
			高➔低	高➔低	高➔低	高➔低	高➔低	高➔低	高➔低	高➔低	高➔低	高➔低	高➔低
			5 4 3 2 1	5 4 3 2 1	5 4 3 2 1	5 4 3 2 1	5 4 3 2 1	5 4 3 2 1	5 4 3 2 1	5 4 3 2 1	5 4 3 2 1	5 4 3 2 1	5 4 3 2 1
1	安寧緩和醫療條例與安寧療護概念	XXX											
2	腎臟緩和醫療的適用條件與預立	XXX											

	醫療自主計畫												
3	腎臟緩和醫療的症狀控制與緩和透析	XXX											
4	腎臟安寧療護實務分享	XXX											
5.	開場與閉幕討論	XXX											

二、課程相關意見:

1.以下四題「請於空格處填入課程編號即可」:

1.1 關於此次課程，請問您對哪一門課最有興趣? (依排名順序填寫前二名) (1)__________ (2) __________

1.2 對於此次課程，您認為若以後舉辦同樣活動時，哪門課是較不需要的? ____

1.3 請問您認為哪門課時間太短 (若無請寫“無”)? (可複選) ______________________________

1.4 請問您認為哪門課時間太長 (若無請寫“無”)? (可複選) ______________________________

2.您參加此次課程的原因? (請勾選) ☐自己的需求 ☐單位的需求 ☐其他______________________________

3.整體而言，您對本次課程的滿意度是如何(包含授課講師、工作人員、講義、場地、硬體設備……等)?

☐非常滿意 ☐很滿意 ☐滿意 ☐不滿意 ☐很不滿意; 理由______________________________

4.在您日常照護腎臟病人的過程中，是否會考慮將腎病緩和醫療或緩和透析的觀念介紹給腎友與其家屬?

☐是 ☐否; 理由______________________________

5.您認為參加本次課程最大收穫是:______________________________

____

6.其他回饋意見: ______________________________

7.您目前服務的單位屬於: ☐醫學中心 ☐區域醫院 ☐地區醫院 ☐基層透析院所 ☐其他______________________________

8.您目前的工作身分(可複選)是屬於: ☐腎臟專科醫師 ☐安寧專科醫師 ☐腎臟科研修醫師(fellow) ☐安寧研修醫師(fellow)

☐腎臟專科病房護理師 ☐血液透析中心護理師 ☐腹膜透析中心護理師 ☐CKD 衛教師 ☐安寧共照管理師 ☐安寧緩和病房護理師

☐其他____

9.服務年資：_____年_____月 (自畢業後開始進入臨床工作/執業算起)
